# An inkblot for beliefs: The Truth Misattribution Procedure

**DOI:** 10.1371/journal.pone.0218661

**Published:** 2019-06-24

**Authors:** Jamie Cummins, Jan De Houwer

**Affiliations:** Department of Experimental Clinical and Health Psychology, Ghent University, Ghent, Belgium; Karl Landsteiner University of Health Sciences, AUSTRIA

## Abstract

An increasing body of evidence shows the importance of accommodating relational information within implicit measures of psychological constructs. Whereas relational variants of the Implicit Association Test (IAT) have been proposed in the past, we put forward the Truth Misattribution Procedure (TMP) as a relational variant of the Affect Misattribution Procedure (AMP) that aims to capture implicit beliefs. Across three experiments, we demonstrate that TMP effects are sensitive to the relational information contained within sentence primes, both in the context of causal stimulus relations of a known truth value (e.g., “smoking causes cancer” vs. “smoking prevents cancer”), as well as in the domain of gender stereotypes (e.g., “men are arrogant” vs. “men should be arrogant”). The potential benefits of the TMP are discussed.

## Introduction

Indirect measurement procedures such as the Implicit Association Test (IAT) are widely used to assess the spontaneous (i.e., automatic) evaluation of stimuli as good or bad[1, 2]. Measurement outcomes that capture such spontaneous evaluations have been referred to as implicit measures of evaluation [3]. Consider the Implicit Association Test (IAT; [4]) as an example of an indirect measurement procedure. The IAT requires participants to quickly categorise target stimuli (e.g., Black and White faces) with valenced attribute stimuli (e.g., positive and negative words) across multiple blocks. Across blocks, the response assignments for these categorisations are varied, such that some blocks require a first response for white and good items and a second response for black and bad items, whereas other blocks require black-good and white-bad categorisations. If the outcome of this procedure is that participants can more quickly categorise White (Black) faces using the same response as positive (negative) words, then this is interpreted as participants showing a spontaneous preference for White faces over Black faces [5].

Another indirect measurement procedure, the Affect Misattribution Procedure (AMP; [6]) is assumed to operate on the principle of misattribution. On each trial, participants are presented with a prime image of either (for example) a Black or White face, which is quickly followed by a Chinese character. Participants are required to evaluate the visual pleasantness of the Chinese character, while ignoring the prime image. In spite of these instructions, participants’ evaluations of the Chinese characters are often influenced by the affective content of the primes that precede them [6, 7]. For example, if a person has a pro-White attitude, then they will be more likely to evaluate characters which follow White faces as positive, compared to when the characters follow Black faces [8]. AMP effects are thought to arise because the spontaneous evaluative reaction that is evoked by the prime stimulus (e.g., a White face) is misattributed to the Chinese character. Because both evaluation and misattribution occur quickly and unintentionally, AMP effects are typically considered to be implicit measures of stimulus evaluation (but see [9] and [10] for a discussion about whether AMP effects are unintentional).

Whereas a standard AMP focuses on stimulus evaluation, research has shown that non-affective, “cold” stimulus features can also be misattributed [11], and other AMP-like procedures have harnessed this in the context of semantic meaning. More specifically, the Semantic Misattribution Procedure (SMP; [12, 13]) is highly similar to the AMP with one critical exception: rather than rating the pleasantness of Chinese characters, participants are asked to speculate about some aspect of the meaning of the characters (e.g., whether it is a male or female name). The SMP has already been used in a variety of contexts for the indirect assessment of psychological constructs. For instance, Ye and Gawronski [14] found that the presentation of a stereotypically-masculine occupation prime word (e.g., doctor) led to participants evaluating Chinese characters as meaning “man” more often than when they were preceded by a stereotypically-feminine prime word (e.g., nurse). The SMP has also been used in the assessment of other constructs, for example sexual preference, personality, self-concept, and risk-taking [13, 15, 16, 17].

The aim of the present paper is to introduce a variant of the SMP that could provide an implicit measure of propositional beliefs. Propositional beliefs are statements that can specify information about the way in which stimuli are related [18]. For example, one could believe that men *are* good, that men *should be* good, or that men *want to be* good. These statements all involve the concepts “men” and “good” but differ with regard to the way in which these concepts are related. The capacity to encode specific relational information sets propositions apart from simple associations that merely link two concepts. Associations differ from propositions also with regard to the fact that only propositions have a truth value; that is, only propositions can be evaluated as being true or false [18]. Most currently available implicit measures have not been designed to capture subtle differences in the way that concepts are related and are thus limited in their capacity to capture specific propositional beliefs. For instance, a standard racial IAT cannot differentiate between whether a particular (Black) participant believes that White people *are* good or whether White people *should be* good. As has been argued elsewhere [19], there are also reasons to suggest that propositional beliefs can arise spontaneously (as opposed to through exclusively deliberative processes), and that there thus is merit in developing measures that capture these implicit (i.e., automatically activated) beliefs.

Aside from attempts at directly modifying the IAT to incorporate relational information (e.g. [20]), two relational IAT-style procedures have been developed: the Implicit Relational Assessment Procedure (IRAP; [21]) and the Relational Responding Task (RRT; [22]); see also [23] for a different type of procedure that can be used to capture propositional beliefs. Both the RRT and the IRAP have shown utility beyond that of the IAT, providing researchers with novel insights into areas such as genital pain [24], body dissatisfaction [25], self-esteem [26], and depression [27]. For instance, Remue and colleagues [27] found that, in the IRAP, depressed individuals showed lower scores for actual self-esteem (i.e., the belief “I *am* good”), as compared to ideal self-esteem (i.e., the belief “I *should be* good”), whereas non-depressed individuals did not show this difference. Within the IAT, on the other hand, depressed and non-depressed individuals showed the same IAT score: a score that would typically be interpreted as revealing positive self-esteem. This illustrates that implicit measures of beliefs can provide additional information above and beyond what standard implicit measures can provide.

In the same way that IAT-like procedures can be adjusted to capture specific propositional beliefs, so too can AMP-like procedures. Consider Ye and Gawronski's [14] earlier-mentioned use of the SMP in the context of gender stereotypes as an example. While Ye and Gawronski found that the prime word “doctor” led to participants being more likely to judge a target as meaning “male” than “female”, it cannot be distinguished whether this was because participants believe that more men than women *are* doctors, or whether they believe that more men than women *should be* doctors (see [28]). Stereotypes are intrinsically relational, in that the tie between the concept and social group is greatly moderated by the stereotypical relationship between them [29, 30]. Traditional versions of the SMP can therefore not capture the full complexity of stereotypes.

We propose a relational variant of the SMP to overcome this issue: the Truth Misattribution Procedure (TMP). The TMP bears parity to the SMP, but with two salient differences. Firstly, primes which are presented are not single images or words; rather, they are sentences presented word-by-word to the participant. Secondly, participants respond to the Chinese characters based on whether the characters mean “true” or “false”. Notionally, prime sentences which are in line with the beliefs of the participant (e.g., “smoking causes cancer”, “safety prevents accidents”) should result in subsequent characters being judged as “true” more often than for those prime sentences which are not in line with the beliefs of the participant (e.g., “smoking prevents cancer”, “safety causes accidents”). Across three experiments in this paper, we seek to provide initial validation for the TMP as a novel measure of implicit beliefs.

## Experiment 1

The first experiment we conducted was an initial exploratory study to assess whether the TMP is capable of capturing differences across trials when the truth value of the prime sentences is objective and normative. For this purpose, we developed three variants of the TMP. The first variant of the TMP was the standard TMP (S-TMP). The S-TMP consists of 100 trials. At the beginning of the procedure, participants were told that there are many symbols in the Chinese language which mean “true” and “false”, and that the goal of the experiment is for them to judge whether each Chinese character means “true” or “false”, while ignoring the prime sentences which precede the characters. In line with the recommendations of De Houwer and Smith [31], participants were also instructed to rely on their gut feelings about the meaning of the characters. On each trial, an initial fixation cross is followed by the word-by-word sequential presentation of a prime sentence. Following this, participants were briefly presented with a target Chinese character, which is then covered by a noisy image. The noisy image remained on-screen until participants gave a response.

The two other variants of the TMP were designed to increase the probability that participants would process the sentence primes on every trial. This should lead to larger effects because a precondition for TMP effects to arise is that participants process the meaning of the prime sentences. For instance, if they only pay attention to the final word of the sentence, rather than the full sentence, it is logically impossible to find (automatic) processing of the truth of the full sentence. With this in mind, in a second variant of the TMP we presented catch trials throughout the procedure which required participants to re-type the full prime sentence, rather than respond to a Chinese character. We will refer to this version as the typing TMP (T-TMP). The third variant, the evaluative TMP (E-TMP), also involved catch trials. However, E-TMP catch trials required participants to respond explicitly to the truth value of the prime sentence, instead of simply re-typing it. We expected that this variant would lead to even greater effect sizes than the T-TMP, given that participants would be induced into a “[truth] evaluation mindset” [23, 32]. On the other hand, it could also be argued that requiring participants to explicitly evaluate the prime sentences might reduce the TMP effect, given that explicitly evaluating primes can lead to clarity about the source of the activated concept, and thus reduce misattribution to the target stimuli [33]. However, the latter conclusion is based on studies which participants responded to *both* the prime and target stimuli within each trial. In our design, participants respond to *either* the prime (in catch trials) or the target (in standard trials) within each trial. As such, we expected that the explicit evaluation of prime sentences on catch trials should not affect the misattribution of truth concepts on standard trials. The main comparison of interest for all versions of the TMP was a comparison of the number of “true” responses to Chinese characters on trials with a valid prime sentence (e.g., “smoking causes cancer”) versus trials with an invalid prime sentence (e.g., “smoking prevents cancer”).

### Method

For all experiments in the current manuscript, ethical approval was provided by the Ethical Committee of the Faculty of Psychology and Educational Sciences at Ghent University (approval numbers 2015/13, 2016/63, and 2016/80). Written consent was obtained from all participants prior to completion of all experiments.

#### Participants

Data for all studies in the current manuscript were collected online via the Prolific Academic website (https://prolific.ac). If participants had previously completed a study using the TMP, they were excluded from participation in subsequent experiments. Given the exploratory nature of this study, we did not conduct an a priori power analysis. However, based on previous research with other misattribution procedures, we aimed to collect approximately 30 participants per condition (i.e., with each of the three variants of the TMP). Given expected attrition rates, we collected data from 100 participants in total. Upon excluding partial or incomplete data (i.e., where participants closed the experiment prior to completing it), 29 participants completed the S-TMP, 19 participants completed the T-TMP, and 21 participants completed the E-TMP. Notably, the drop-out rate for both the T-TMP and E-TMP was much higher than for the S-TMP. This was likely due to the fact that the T-TMP and E-TMP are more demanding for participants to complete, as two different task-types are interspersed. Across all three experiments in the current paper, higher drop-out rates for the E-TMP, and in particular the T-TMP, were seen, as compared with the S-TMP. Completed data from 69 participants (40 women, 29 men) was collected (M age = 33.3 years, SD = 10.4). All participants were paid £1.50 for completing the study, based on an expected completion time of 15 minutes.

#### Experimental design

We employed a mixed between-within experimental design. There was one between-subjects factor, TMP-variant completed, with three levels: S-TMP, T-TMP, and E-TMP. As well as this, there was one within-subjects factor, the truth value of the prime which preceded the Chinese character on each trial on the TMP, with two levels: true prime and false prime.

#### Measures and procedure

All experimental materials were programmed in Inquisit 4.0 (Millisecond software) and were presented using the Inquisit Web Player. Each variant of the TMP consisted of ten true sentences and ten false sentences as stimuli (see Appendix 1 for the specific stimuli used). All sentences consisted of three words, in the form of noun-verb-noun, where the verb specified some causal relation between the two nouns. These sentences were taken from a normed database of causal stimulus relations [34]. True sentences consisted of five “causes” and five “prevents” statements taken from the database. False sentences were constructed through taking each true sentence and changing the relational qualifier to its opposite (e.g., “smoking causes cancer” becomes “smoking prevents cancer”). All participants provided basic demographic information, before completing one of three variants of the TMP. Following this, participants also answered a number of exploratory self-report questions relating to their experience of the procedure.

All TMP variants consisted of 100 trials in total. In the S-TMP, all 100 trials consisted of ‘standard’ trials, with each of the 20 prime sentences presented randomly 5 times. These began with the presentation of a fixation cross on-screen for 500ms, followed by the sequential presentation of the prime sentence, one word after the other, each word for 200ms. Note that whereas the length of this presentation for the prime stimulus is longer than the typical length of time for prime presentation in AMP/SMP studies (which usually present primes for only 75-100ms), we opted for this presentation length in order to allow sufficient time for participants to process the semantic content of the sentences, in line with previous research using a similar paradigm (Wiswede et al., 2012). Following this, the screen cleared for 100ms. A Chinese character was then presented for 100ms, before being covered by a mask. The mask remained on-screen until the participant responded, at which point the fixation cross for the next trial immediately appeared on-screen. Participants were instructed to respond based on whether they believed the Chinese character meant “true” or “false”, and to ignore the prime sentence which preceded each character. Across all TMP variants, the position of the “true” and “false” keys was counterbalanced across participants. Half of the participants were instructed to press the “E” key for true, and the “I” for false, whereas the other half were instructed to press the “I” key for true and the “E” key for false.

In the T-TMP, 70 trials were standard trials. The remaining 30 trials were ‘typing-catch’ trials. The 20 different prime sentences were each presented randomly 5 times across the 100 total trials from the two trial types. For the catch trials, the prime sentence was presented as on standard trials. However, rather than presenting a Chinese character following the prime sentence, typing-catch trials involved presenting a textbox on-screen, along with a prompt which instructed participants to re-type the sentence which had just been presented. Participants were provided with either ‘correct’ or ‘wrong’ feedback after completing each of these trials based on whether they had correctly re-typed the prime sentence or not (participants were informed that they were required to spell the sentences correctly, and that misspellings of words would lead to ‘wrong’ feedback). Like the T-TMP, in the E-TMP, 70 trials were standard trials, and 30 trials were catch trials, with the 20 prime sentences presented randomly 5 times across the two trial-types. The catch trials of the E-TMP, however, were ‘evaluation’ rather than ‘typing’ trials. For these trials, the prime sentence was again presented as on a standard trial. However, rather than presenting a Chinese character following the prime sentence, participants were presented with the prompt “??-True or false-??”. Participants were instructed at the beginning of the procedure that, upon seeing this prompt, they were required to intentionally evaluate the truth of the prime sentence (using the same “E” and “I” key presses as used in the implicit trials). Participants were also given ‘correct’ or ‘wrong’ feedback based on the accuracy of their response.

### Results

We firstly sought to investigate whether there was a TMP effect (i.e., larger number of “true” responses when Chinese characters were preceded by true sentences than false sentences) for each of the three TMP variants. In order to assess this, we used two analytic strategies for each of the three variants. We firstly conducted the ‘standard’ analysis: within-subjects t-tests, with proportion of “true” responses as DV, and prime type (true or false sentence) as IV. As well as this, given our small sample size, we also constructed logistic mixed-effects models for each of the three variants, with Response as DV (with “false” responses coded as 0, and “true” responses coded as 1; coded identically in all subsequent experiments), Prime Type as IV (with “false” primes coded as 0, and “true” primes coded as 1; coded identically in all subsequent experiments), and Participant ID modelled as a random effect, in order to answer this question with a more statistically-powerful method (see Table 1 for results from these analyses). In the t-tests, there was a significant difference in proportion of ‘true’ responses between trials in the E-TMP, but not the T-TMP or S-TMP. However, in the LMMs, all three variants showed a significant TMP effect. Additional LMMs revealed that there was no effect of the valence of the final prime word (e.g., “cancer” in “smoking causes cancer” may be considered of negative valence) on responses in the TMP (all *p*s > .05). We next investigated whether effect sizes varied across each of the TMP variants. In order to do this, we conducted a 2 (Prime Type: True or False) x 3 (TMP Variant: S-TMP, T-TMP, or E-TMP) mixed between-within groups ANOVA, with proportion of ‘true’ responses as DV. This analysis did not reveal a main effect of TMP Variant on the number of ‘true’ responses to characters, *F*(1, 134) = .378, p = .54, ω^2^ = 0.003, but there was a significant main effect of Prime Type, *F*(1, 134) = 31.51, p < .001, ω^2^ = 0.171. Most critically, we found a significant interaction between Prime Type and TMP Variant on proportion of correct responses, *F*(1, 134) = 11.91, p < .001, ω^2^ = 0.061. Subsequent pairwise comparisons yielded significant differences in TMP effects between the E-TMP and the S-TMP, *t*(98) = 4.56, *p* < .001, d = 0.39, and between the E-TMP and the S-TMP, *t*(78) = 3.62, p < .001, d = 0.77. No such difference was found between the T-TMP and the S-TMP *t*(94) = 0.21, p = 0.84, d = 0.20.

**Table 1:** Results (t-test and LMMs) for each of the three TMP variants in Experiment 1.

TMP variant	Mean proportion of “true” responses		*t*-test			Logistic mixed-effects model	
	True prime (SD)	False prime (SD)	*t*-value	*p*-value	Cohen’s d	Odds ratio (95% CIs)	*p*-value
S-TMP	.58 (.22)	.46 (.20)	1.72	0.10	0.32	1.65 (1.43, 1.92)	< .001
T-TMP	.57 (.27)	.47 (.29)	0.85	0.41	0.20	1.53 (1.23, 1.90)	< .001
E-TMP	.78 (.16)	.32 (.23)	6.01	< .001	1.31	8.16 (6.41, 10.40)	< .001

### Discussion

Results from Experiment 1 were broadly consistent with our expectations: effects were seen in the LMMs for each of the three TMPs in the expected direction, and the TMP effects were largest for the E-TMP and smallest for the S-TMP. However, only the E-TMP showed a significant effect of prime type in the standard t-tests. Given that the LMMs represent a highly similar, but statistically more powerful, analysis to the t-tests, one could argue that we simply did not power our experiment sufficiently to detect true effects for the S-TMP and T-TMP. We therefore opted to conduct a further experiment, Experiment 2, with a larger sample, in order to verify our findings using more adequately-powered analyses.

## Experiment 2

For Experiment 2, we intended to replicate the design of Experiment 1, and to simply collect data for more participants. However, we also made a first effort in examining the implicitness of TMP effects. Prime stimuli in the TMP are presented for a much longer duration (600ms) than prime stimuli in the AMP or in the SMP (~100ms). Whereas previous research with the AMP has shown that AMP effects persist even when prime stimuli remain on-screen for as long as 1500ms [6], it is unclear whether the mechanism for effects at this presentation time remains based on unintentional misattribution. Notionally, there is a possibility that effects in misattribution procedures with elongated prime presentation times are the consequence of intentional responding (i.e., participants deliberately using their truth evaluation of the prime sentences as a source for responding to the targets) rather than unintentional responding. Given that the purported implicitness of misattribution-style procedures resides in their ability to capture unintentional responses, examining this issue is vital for validating the implicit nature of TMP effects [3].

Fortunately, previous research using the AMP has provided methods to assess the relative intentionality of responding within the procedures. For instance, Bar-Anan and Nosek [9] and Payne and colleagues [10] investigated intentional responding through the use of self-report measures. Experiment 1 of Payne et al. in particular provides a clear means of addressing this question. After completing the AMP, participants were asked one of two questions: either whether their responses to targets were *intentionally* based on the affective value of the primes, or whether their responses to targets were *unintentionally* influenced by the affective value of the primes. Payne et al. argued that if effects in the AMP are the result of intentional responding, then higher self-reported rates of intentional influence should predict greater AMP effects, *and* higher self-reported rates of unintentional influence should *not* predict greater AMP effects. If both self-reported intentional and unintentional influence predict AMP effect sizes (as was found by Payne et al.), then it can be inferred that participants are unable to discriminate the source by which their responses were influenced, and that they are confabulating their reasoning post-hoc, evidencing the unintentional nature of responding within the AMP [35]. As such, we employed the method of Payne et al. here, in order to discern the extent to which effects in each of the three TMP variants can be considered unintentional (and thus, in that sense, implicit). For all subsequent experiments in this paper (i.e., Experiments 2 and 3), we report how we determined our sample size, all data exclusions (if any), all manipulations, and all measures in the study.

### Method

#### Participants

Based on pre-registered power analyses (see https://osf.io/v28gm/) we determined that we would collect completed data from a minimum of 360 participants in total. We collected (partial or complete) data from 420 total participants. After exclusions, our final sample (*n* = 396) consisted of 201 men, 193 women, 1 person who identified as agender, and 1 participant with no gender given, with a mean age of 36.36 years (SD = 12.26). 140 participants completed the S-TMP, 118 completed the T-TMP, and 138 completed the E-TMP. Based on an expected completion time of 15 minutes, all participants were paid £1.50 for their participation.

#### Materials and procedure

All materials were programmed in Inquisit 4.0 and presented using the Inquisit Web Player. Measures employed were identical to those of Experiment 1, with one important addition: two questions following the completion of the TMP, one relating to the intentional influence of primes on responding within the task, and the other relating to the unintentional influences of primes on responding. The wording of these questions was identical to those presented by Payne and colleagues [10], with the exception that they were adjusted to be oriented on semantic content of Chinese characters/primes, rather than valence. For the intentional question, participants were asked “When you had to rate the meaning of the Chinese characters, did you intentionally rate the truth value of the sentences instead?”. For the unintentional question, participants were asked “Did the truth value of the sentences unintentionally influence your ratings of the meaning of the Chinese characters?”. Whereas Payne et al. presented only one of these questions to each participant and conducted their analysis at the between-subjects level, we administered both questions to all participants. However, we counterbalanced the order of the presentation of the questions, so that we could also replicate Payne et al.’s analysis.

### Results

#### Pre-registered

We firstly attempted to replicate the three within-subjects t-tests from Experiment 1, that is, one for each of the three TMP variants, comparing the proportion of ‘true’ responses for true primes versus false primes. Note that we did not preregister the use of logistic mixed-effects modelling here, in contrast to our analytic strategy in Experiment 1, because we powered our design in such a way to detect the expected effect sizes using the basic t-test analyses. However, unsurprisingly, applying the more powerful LMMs to the Experiment 2 data yielded the same significant effects as the t-tests reported in the manuscript. There was a significant TMP effect for all three TMP variants (see Table 2).

**Table 2:** Results (t-tests) for each of the three TMP variants from Experiment 2.

TMP-variant	Mean proportion of “true” responses		*t*-test		
	True prime (SD)	False prime (SD)	*t*-value	*p*-value	Cohen’s d
S-TMP	.56 (.19)	.43 (.19)	4.70	< .001	0.40
T-TMP	.62 (.29)	.39 (.29)	4.46	< .001	0.41
E-TMP	.72 (.19)	.32 (.21)	13.26	< .001	1.13

Next, we attempted to replicate the second finding of Experiment 1, more specifically that TMP effect sizes differed across procedures. For this, we used the 2 x 3 mixed ANOVA, as in Experiment 1. We did not observe a main effect of TMP Variant on proportion of ‘true’ responses, *F*(1, 788) = 1.73, *p* = .19, ω^2^ = 0.001, but we did observe a significant main effect of Prime Type, *F*(1, 788) = 245.72, *p* < .001, ω^2^ = 0.226. Most importantly, we replicated the significant interaction effect between Prime Type and TMP, *F*(1, 788) = 47.30, *p* < .001, ω^2^ = 0.043. Pairwise comparisons for the interaction showed that the E-TMP and the S-TMP effect sizes differed significantly, *t*(554) = 9.06, *p* < .001, d = 0.77, as well as the E-TMP and T-TMP effect sizes, *t*(510) = 4.41, *p* < .001, d = 0.39, and the T-TMP and S-TMP effect sizes, *t*(514) = 2.28, *p* = 0.02, d = 0.20.

We next sought to address the question of intentionality. Firstly, we intended to replicate the analysis of Payne et al., that is, to show that at the between-subjects level, the self-reported effect of influence was predicted by TMP effect size, but not the interaction between TMP Effect Size and Question Wording (Unintentional vs. Intentional; Unintentional coded as 0, Intentional coded as 1, coded similarly across all subsequent analyses in all Experiments), for each of the three TMP variants. For this, as in Payne et al., we used a regression analysis for each of the three TMP variants, with Self-Report Score as DV, and Question Wording and TMP Effect Size as IVs. For the S-TMP, we did not observe a main effect of either TMP Effect Size (*β* = 0.39 [95% CI: -0.14, 0.92], *p* = .15) or Question Wording (*β* = 0.07 [95% CI: -0.11, 0.24], *p* = .44) in predicting Self-Report Score. The interaction between TMP Effect Size and Question Wording was also not significant (*β* = -0.11 [95% CI: -0.64, 0.42], *p* = .69). For the T-TMP however, the opposite pattern was seen: there were main effects for both TMP Effect Size (*β* = -1.20 [95% CI: -1.72, -0.66], *p* < .001) and Question Wording (*β* = -0.37 [95% CI: -0.57, -0.16], *p* < .001), as well as a significant interaction between these variables (*β* = 1.59 [95% CI: 1.02, 2.16], *p* < .001). For the E-TMP, a similar trend to that of the S-TMP was seen: no main effects for either TMP Effect Size (*β* = -0.03 [95% CI: -0.55, 0.49], *p* = .90) or Question-Wording (*β* = -0.11 [95% CI: -0.36, 0.13], *p* = .37), as well as no interaction between them (*β* = 0.29 [95% CI: -0.26, 0.85], *p* = .30).

Because each participant answered both the intentionality and unintentionality questions, we could also examine the question of intentionality by testing whether TMP effect sizes are predicted by self-reported intentional responding *above and beyond* unintentional responding. We employed two-step hierarchical regression models for each of the TMP variants. The first step of each model involved investigating whether Unintentionality scores predicted TMP Effect Sizes. The second step of each model involved adding in Intentionality scores, and the interaction between Intentionality and Unintentionality scores. For the S-TMP, Unintentionality predicted TMP Effect Sizes in Step 1, (*β* = 0.24 [95% CI: 0.08, 0.4], *p* = .004). In the second step of the model, Unintentionality no longer predicted TMP Effect Sizes, and nor did either of the two other factors (1 > *p*s > .11). For the T-TMP, Unintentionality was not a significant predictor in Step 1 (*β* = 0.15 [95% CI: -0.03, 0.33], *p* = .11). In the second step, Unintentionality remained non-significant, and none of the other factors were significant predictors (.90 > *p*s > .61). For the E-TMP, Unintentionality was a significant predictor in the first step of the model (*β* = 0.22 [95% CI: 0.05, 0.38], *p* = .01). In the second step, Unintentionality remained a significant predictor (*β* = 0.47 [95% CI: 0.05, 0.88], *p* = .03) but both Intentionality (*β* = 0.74 [95% CI: 0.37, 1.12], *p* < .001) and the interaction between Intentionality and Unintentionality (*β* = -0.68 [95% CI: -1.3, -0.05], *p* = .04) added to the predictions of the model, above and beyond Unintentionality alone.

Finally, we sought to determine whether self-reported influence varied as a function of the interaction between question-wording and TMP variant. To address this, we conducted a 3 (TMP Variant) x 2 (Question-Wording) mixed ANOVA with Self-Reported Influence as DV. Most crucially, we did not find an interaction effect between Question-Wording and Condition, *F*(1, 788) = 1.18, *p* = .28, ω^2^ = 0.000. There was a significant main effect of Condition on Self-Report Scores, *F*(1, 788) = 40.43, *p* < .001, ω^2^ = 0.047, and there was no main effect of Question-Wording, *F*(1, 788) = 0.55, *p* = .46, ω^2^ = 0.001.

#### Not preregistered

In addition to the above pre-registered analyses, we also conducted an additional unregistered analysis: the assessment of the reliability of the three variants of the TMP. To assess reliability, we calculated odd-even split-half reliability with Spearman-Brown correction for each of the three variants. Reliability for each of the three variants was very high (S-TMP *Rsb* = .94; T-TMP *Rsb* = .97; E-TMP *Rsb* = .93).

### Discussion

TMP effects were seen in each variant but effect sizes varied as a function of the variant, with the S-TMP again showing the smallest effect and the E-TMP again showing the largest effect. Notably, the increased power in the design of Experiment 2 relative to Experiment 1 resulted in effects being seen in each TMP variant not only in the LMMs, but also in the standard t-tests. As well as this, analyses indicated that there was no influence of intentional responding on the S-TMP. This finding of the unintentionality of the S-TMP is further bolstered by the fact that, for the other two TMP variants, we did find effects of intentionality. For the T-TMP, rates of self-reported intentional responding increased as a function of T-TMP effect size, while unintentional responding did not. For the E-TMP, intentional responding predicted E-TMP effect sizes above and beyond unintentional responding. These findings thus indicate that both the T-TMP and E-TMP are susceptible to intentional responding. However, this criticism may be less salient for the E-TMP than the T-TMP, given that: (i) the effect of intentionality was not seen for the E-TMP in the standard analytic strategy used to assess effects of intentionality in misattribution procedures, (ii) in the analysis which showed a relatively strong influence of intentionality for the E-TMP, unintentional influence also contributed significantly in predicting scores, (iii) in this analysis, unintentional and intentional influence also interacted in predicting scores, and (iv) the largest effect sizes were seen in the E-TMP. From these points, although there may have been a relatively strong degree of intentional influence over responding in the E-TMP, it may be argued that this is also balanced with a strong unintentional aspect, and is also offset by virtue of the fact that effect sizes in the E-TMP are generally largest (an increase in size which cannot be solely attributable to the presence of intentional responding, given that a similar magnitude was not seen in the T-TMP, where intentionality is strongly present).

## Experiment 3

Experiments 1 and 2 provided evidence for the effectiveness of the TMP in demonstrating effects when using normed causal stimulus relations. Experiment 3 sought to extend the development of the TMP by providing evidence for its ability to capture differences in stimulus relations in a more socially relevant context, specifically, in the context of gender stereotypes. We opted for the domain of gender stereotypes for two reasons. Firstly, the SMP has formerly been used successfully in this context, indicating that misattribution procedures are likely suitable for this domain [14]. Secondly, the conceptualisation of “stereotypes” when assessed by associative measures in general (the IAT, SMP, etc.) is relatively simplistic: an association between a social group and a specific trait [36]. However, as we discussed earlier, this conceptualisation is not wholly accurate. Gender stereotypes can be present in descriptive terms (e.g., the belief that women *are* gentle, and that men *are* aggressive). However, such stereotypes can also involve other types of relations, such as what members of a gender are supposed to do (i.e., prescriptive terms such as the belief that women *should be* gentle and that men *should be* aggressive). Accommodating these relational features is crucial for researchers to fully understand the dynamics of stereotypy. For example, hostility towards women in the workplace is considered to be based on a discrepancy between the actual behavior of women (e.g., women who *are* dominant in the workplace) and prescriptive-stereotypes towards women (women *should be* submissive in the workplace [28, 37]). This distinction between descriptive and prescriptive gender stereotypes has been closely-studied in a variety of contexts within the gender stereotypes field [38]. As such, the TMP’s unique status as a relational misattribution measure may be of particular interest to researchers in this field, as it can very easily parse between descriptive (“are”) and prescriptive (“should”) gender stereotype relations. Verifying the TMP’s ability to show differences based on the specific relational type of a sentence oriented on gender stereotypes therefore represents an important first step in this regard.

Given the finding of intentional responding in the T-TMP from Experiment 2, we opted to exclude its use from the current experiment. However, we included the use of both the S-TMP (due to the lack of effect of intentionality in Experiment 2) and the E-TMP (given the advantages associated with its use which arguably outweigh the partial impact of intentional responding). We intended to examine whether both variants of the TMP (i) are capable of distinguishing between descriptive and prescriptive gender stereotype relations, (ii) are capable of distinguishing descriptive stereotypes between men and women, and (iii) are not susceptible to intentional influence.

### Method

#### Participants

Based on pre-registered power analyses (see https://osf.io/rfts9/) we determined that we would collect data from a minimum of 240 women. As specified in our preregistration, we opted to collect from women only as they exhibited larger explicit stereotyping effects in our normative index than men. We collected data from 274 participants. After exclusions, our final sample consisted of 253 women with a mean age of 39.06 years (SD = 11.79). 126 participants completed the S-TMP, and 127 completed the E-TMP. Based on an expected completion time of 12 minutes, all participants were paid £1.05 for their participation.

#### Experimental design

The design of the study was identical to Experiment 2, with two exceptions. First, only the S-TMP and E-TMP were used. Second, after completion of the TMP, participants also provided explicit ratings of their agreement with each of the gender stereotype statements which were used within the TMP. For these explicit ratings, participants were presented with each statement and asked to indicate, on a 9-point Likert scale, the extent to which they agreed with that statement.

#### Materials and measures

All materials were programmed in Inquisit 4.0 and presented using the Inquisit Web Player. Measures employed were identical to those of Experiment 2, with two variations. First, primes in the current experiment consisted of sentences oriented on the relation between men/women and specific traits (e.g., “men are arrogant”, “women should be mature”). Trait stimuli were chosen based on a normative index collected by the authors (see https://osf.io/7depg/), with the following criteria: (i) large differences in how the trait is evaluated between “men are” and “men should be”, (ii) large differences in how the trait is evaluated between “men are” and “women are”, (iii) an equal number of desireable and undesireable traits. Traits were defined as “desireable” or “undesireable” based on whether their mean “should be” scores for both men and women were above or below 5 on the 9-point Likert scale used in the normative index. Four trait stimuli in total were selected: two desireable (mature, express emotion), and two undesireable (child-like, arrogant). Based on the normative index, men were evaluated as being more like the undesireable traits than women, while women were rated as being more like the desireable traits than men. As well as this, men were evaluated as being more like the undesireable traits than they should be, and were evaluated as being less like the desireable traits than they should be (see Appendix 2 for the specific stimuli used). A second variation was that, in the E-TMP, participants no longer received “correct” or “wrong” feedback when they responded on evaluative-catch trials. We eliminated this feedback because participants might differ in their personal truth evaluation of stereotype related statements.

### Results

#### Preregistered

Table 3 presented the means and standard deivations for the proportion of “true” responses to each of the prime types used in our analyses here. We first hypothesised that there would be oppositional TMP effects for Desireable and Undesireable traits based on different relation types for men, such that ‘men-are-undesireable’ sentences would lead to more “true” responses than ‘men-are-desireable’ sentences, while ‘men-should-be-desireable’ sentences would lead to more “true” responses than ‘men-should-be-undesireable’ sentences.

**Table 3:** Proportions of “true” responses” for each of the beliefs assessed in the TMP in Experiment 3.

TMP-variant	Mean proportion of “true” responses					
	Men are desireable	Men are undesireable	Women are desireable	Women are undesireable	Men should be desireable	Men should be undesireable
S-TMP	.57 (.22)	.44 (.21)	.61 (.22)	.42 (.21)	.58 (.22)	.39 (.22)
E-TMP	.58 (.26)	.45 (.26)	.7 (.27)	.35 (.25)	.68 (.27)	.34 (.26)

In order to investigate this, we preregistered and employed two analyses for both the S-TMP and the E-TMP. Firstly, we used linear mixed-effects model for each of the two procedures, in order to investigate the presence of a Desireability x Relation Type interaction for male-gender trials on responses to the Chinese characters in the TMP. In all cases where Desireability is used, Desireable traits are coded as 0, and Undesireable traits are coded as 1. Within Relation Type, Are relations are coded as 0, and Should relations are coded as 1. For clarity, we describe a number of models used in this experiment in Wilkinson notation. Wilkinson notation is a popular method of describing models without the need to specify coefficient values, and is essentially identical to the R syntax used to produce these models (for further reading, see [39]). In Wilkinson notation, this model may be described as:

Response ~ Desireability * Relation Type + (1 | Participant)

A significant interaction effect was seen for both the S-TMP (OR = 0.74, 95% CI [0.61, 0.88], *p* = .001) and the E-TMP (OR = 0.43, 95% CI [0.34, 0.54], *p* < .001). Secondly, at the participant level of analysis, we produced linear regression models for both TMP variants. For the S-TMP, there was no significant Desirability x Relation Type interaction, (*β* = -0.07, 95% CI [-0.27, 0.01], *p* = .06). For the E-TMP, there was a significant interaction (*β* = -0.31, 95% CI [-0.44, -0.17], *p* < .001).

We next investigated the hypothesis that there would be oppositional TMP effects between men and women for descriptive relations, based on the two levels of desirability. In order to investigate this, we again computed two models for each variant of the TMP: one LMM, and one regression. The LMM constructed for this analysis can be expressed as:

Response ~ Desireability * Sentence Gender + (1 | participant)

For Sentence Gender, Male Sentence Gender is coded as 0, and Female Sentence Gender is coded as 1. In the LMMs, there was a significant interaction between Sentence Gender and Desireability for both the S-TMP (OR = 0.72, 95% CI [0.60, 0.87], *p* < .001) and the E-TMP (OR = 0.39, 95% CI [0.31, 0.49], *p* = .001), indicating that participants were more likely to respond “true” when Desireable traits were preceded by “women are” compared to when they were preceded by “men are”, but this trend was reversed when the trait was Undesireable. For the linear regressions, no significant interaction was found for the S-TMP (*β* = -0.14, 95% CI [-0.28, 0], *p* = .056), while a significant interaction was found for the E-TMP (*β* = -0.32, 95% CI [-0.45, -0.18], *p* < .001).

Finally, we sought to investigate the question of whether responses in the TMP were unintentional. To do this, we again conducted the analysis of Payne and colleagues [10], as in Experiment 2. However, one important difference between the current experiment and Experiment 2 resides in the fact that the gender stereotypes version of the TMP could produce multiple different TMP effects: for instance, the difference between the men-are TMP score and the men-should TMP score; the difference between the men-are TMP score and the women-are TMP score; etc. Given this variety of potential TMP effects, we opted to conduct the intentionality analysis for both the men-are vs. men-should TMP effect, and the men-are vs. women-are TMP effect (i.e., the two differences which were of interest in our previous analyses in the current experiment) for both the S-TMP and the E-TMP. We expected that we would not find a significant interaction between explicit Question-Wording and TMP Effect Sizes in predicting Self-Report Scores in any of the analyses.

For the S-TMP with the are-vs-should TMP effect, there was a main effect of TMP Effect (*β* = 0.34, 95% CI [0.12, 0.56], *p* = .003) for predicting Self-Report Score. There was no main effect for Question Wording (*β* = 0.03, 95% CI [-0.15, 0.20], *p* = .77). Most importantly, we did not find an interaction between TMP Effect and Question Wording (*β* = -0.11, 95% CI [-0.33, 0.11], *p* = .34). For the S-TMP with the women-vs-men TMP effect, a similar pattern was demonstrated: there was a main effect of TMP Effect (*β* = 0.33, 95% CI [0.11, 0.55], *p* = .003), no main effect for Question Wording (*b* = 0.03, 95% CI [-0.14, 0.20], *p* = .75), and no interaction between TMP Effect and Question Wording (*β* = -0.14, 95% CI [-0.35, 0.08], *p* = .22). For the E-TMP with the are-vs-should TMP effect, there was a main effect of TMP effect on Self-Report Scores (*β* = 0.32, 95% CI [0.09, 0.54], *p* = .006). No main effect of Question-Wording was found (*β* = 0.07, 95% CI [-0.12, 0.26], *p* = .46). Once again, we did not find an interaction between TMP Effect and Question Wording (*β* = -0.14, 95% CI [-0.37, 0.09], *p* = .25). Finally, for the E-TMP with the women-vs-men TMP effect, we found a main effect for TMP Effect Size (*β* = 0.34, 95% CI [0.12, 0.57], *p* = .003). We again did not find either a main effect of Question-Wording (*β* = 0.10, 95% CI [-0.09, 0.29], *p* = .28), nor an interaction effect between TMP Effect Size and Question-Wording (*β* = -0.18, 95% CI [-0.41, 0.05], *p* = .12), on Self-Report Scores.

#### Not preregistered

We originally preregistered the use of only the intentionality analysis of Payne and colleagues [10]. However, based on a recommendation from one reviewer, we also conducted the additional intentionality analysis which we also used in Experiment 2 (i.e., determining whether intentionality predicts TMP scores above and beyond unintentionality), using both TMP scores calculated from both TMP variants. For the S-TMP with the are-vs-should TMP effect, in the first step of the model Unintentionality was a significant predictor of TMP effect size, (*β* = 0.27, 95% CI [0.10, 0.44], *p* = .002). In the second step of the model (adding in Intentionality), neither Intentionality, Unintentionality, nor their interaction were significant in predicting TMP effect sizes (.84 > *p* > .088). For the S-TMP with the men-vs-women effect, Unintentionality was again a significant predictor in the first step of the model (*β* = 0.25, 95% CI [0.08, 0.42], *p* = .005). In the second step of the model, none of the model terms significantly predicted TMP effect sizes (.75 > *p* > .43). For the E-TMP with the are-vs-should effect, Unintentionality significantly predicted TMP effect sizes, (*β* = 0.23, 95% CI [0.06, 0.40], *p* = .009). The second step of the model once more led to all terms not reaching significance, .62 > *p* > .36. Finally, for the E-TMP with the men-vs-women effect, Unintentionality was once more a significant predictor of TMP effect sizes (*β* = 0.22, 95% CI [0.05, 0.39], *p* = .013), and in the second step of the model all terms were not significant (.84 > *p* > .32). Across both scores for both TMPs, then, Intentionality did not predict TMP effect sizes above and beyond Unintentionality.

Prompted by one reviewer’s comments, we sought to explore the relationship between TMP effects produced by (i) standard trials and (ii) evaluative catch trials, as well as their relationship to explicit truth ratings provided at the end of the experiment. We calculated TMP effects from the evaluative catch trials in the same way as TMP effects (i.e., the difference between proportions of “true” responses across different trial types) with “direct” effects (i.e., based on directly evaluating the prime stimulus’ truth value) calculated on the basis of the truth evaluation catch trials and “indirect” effects (i.e., when attempting to evaluate the truth value of the Chinese character targets) calculated on the basis of the standard trials. In order to investigate the relationship between these measures, we examined the correlations between all measures, in a similar fashion to Payne and colleagues [40], who investigated a similar question using a modified AMP which also allowed for the calculation of direct and indirect AMP effects. Tables 4 and 5 illustrate these relationships the relationships between scores on all measures for (i) are-vs-should scores, and (ii) men-vs-women scores, respectively.

**Table 4:** Correlations between measures for scores based on “are-vs-should” relations. All *p*s for correlations < .001.

	Indirect TMP effect	Direct TMP effect	Explicit
Indirect TMP effect	1		
Direct TMP effect	.51 (.37 - .63)	1	
Explicit	.35 (.18 - .49)	.43 (.27 - .56)	1

**Table 5:** Correlations between measures for scores based on “men-vs-women” relations. All *p*s for correlations < .001.

	Indirect TMP effect	Direct TMP effect	Explicit
Indirect TMP effect	1		
Direct TMP effect	.60 (.47 - .70)	1	
Explicit	.37 (.21 - .51)	.45 (.30 - .58)	1

### Discussion

Results from Experiment 3 in general were supportive of our hypotheses. Both the S-TMP and the E-TMP showed divergent effects between relation types for desireable/undesireable traits for men: participants implicitly endorsed beliefs involving men and desireable traits more than men and undesireable traits when the relation type was prescriptive (i.e., “men *should be* mature” primes lead to rating the characters as true more often than “men *should be* arrogant” primes), but showed the opposite trend when the relation type was descriptive (i.e., “men *are* arrogant” primes lead to rating characters as true less frequently than “men *are* mature” primes). A similar expected divergence in effects was found between the gender within the sentences for desireable/undesireable traits with descriptive relations: when the gender was female, primes with desireable traits lead to characters rated as true more often than primes with undesireable traits (i.e., “*women* are mature” primes lead to judging characters as meaning true more frequently than “*women* are arrogant”); when the gender was male, the opposite pattern was seen (i.e., “*men* are arrogant” prime sentences lead to more true ratings of characters than “*men* are mature”). These results suggest that the TMP is sensitive to the content of the prime sentences, and most saliently, the relational information contained within these primes. In addition, no evidence of intentional influence was found in either the S-TMP or the E-TMP using either Payne and colleagues’ [10] analysis, or using our novel intentionality analysis, suggesting that the measures are capable of capturing unintentional responding (and therefore are implicit in this sense).

## General discussion

In this paper, we sought to introduce, and provide initial validation for, a novel misattribution procedure capable of capturing effects of relational information: the Truth Misattribution Procedure (TMP). In Experiment 1, we found preliminary evidence to suggest that effects in three variants of the TMP (the S-TMP, T-TMP, and E-TMP) reflected the truth value of prime sentences. Experiment 2 provided a preregistered replication of the findings of Experiment 1. Experiment 2 also suggested that the T-TMP was susceptible to intentional responding to the prime sentences, the E-TMP was susceptible to a lesser extent, while the S-TMP was not susceptible. Experiment 3 (also preregistered) provided further validation of the S-TMP and the E-TMP in the context of gender stereotypes: participants generally evaluated Chinese characters which followed stereotype-consistent sentences (e.g., men are arrogant; women are mature; men should be mature) as true more often than when the characters followed stereotype-inconsistent sentences (e.g., men are mature; women are arrogant; men should be arrogant).

The capacity of the TMP to distinguish between, and quantify simultaneously, both descriptive and prescriptive stereotypes exceeds the exclusively associative-like stereotypes which the SMP can measure. To further illustrate the utility of this measure in the context of gender stereotypes, consider the following example. When presented with two individuals, Richard and Jennifer, and asked who is more likely to be a scientist and who is more likely to be an artist, people will generally believe at the explicit level that both individuals are equally likely to be either profession (i.e., the “fairness principle”). At the implicit level, however, people tend to believe Richard is more likely to be the scientist, and Jennifer the artist (i.e., the “base rate” principle). When presented with individuating information that is counter to the base rate principle (e.g., Richard is the artist), people’s implicit beliefs do not change accordingly, although their explicit beliefs do [41, 42]. While this finding is interesting, it is unclear which specific stereotype belief(s) are unchanged. It could be the case, for example, that the implicit descriptive belief that “Richard is an artist (rather than a scientist)” in fact does change in accordance with the individuating information presented; however, the implicit prescriptive belief that “Richard *should be* a scientist (rather than an artist)” may persist, and ultimately override any change in the descriptive stereotype. If it is in fact the persistence of implicit prescriptive stereotypes, in spite of changes in descriptive stereotypes, which leads to no change in IAT scores after individuating information is known, then it is not the case that “implicit beliefs uphold base rates and appear relatively impervious to counterstereotypic facts”, as Cao and Banaji [40] argue. Rather, it may be that descriptive beliefs change in accordance with counterstereotypic facts, while prescriptive beliefs perseverate in spite of them. Ultimately, the question of which implicit beliefs specifically change/do not change can only be approached using indirect measurement procedures which can capture relational information. As such, the TMP is well-placed to address this question with greater depth and specificity.

Notably, the findings from the current study add to the growing body of evidence which supports a propositional view on implicit processing according to which representations involved in implicit processes are propositional, rather than associative, in nature [19, 43]. In Experiments 1 and 2, the inversion of causal relations in true sentences (e.g., changing “causes” to “prevents” in the sentence “smoking causes cancer”) led to an according inversion of effects in the TMP, whereas in Experiment 3, changes in the stereotype-relation in prime sentences also led to according changes in TMP effects (e.g., “men are arrogant” led to evaluation of characters as true, while “men should be arrogant” led to evaluation of characters as false). All other factors in these sentences remained constant; yet, differing relational qualifiers in the prime sentences led to differing effects at the implicit level. The implicitness of these effects in the S-TMP was supported by virtue of the fact that self-report scores were not predicted by the interaction between S-TMP effect sizes and whether the phrasing of the question was a rating of intentionality vs. unintentionality, and that intentionality ratings did not predict TMP sizes above and beyond unintentionality ratings (though the former was the case for the T-TMP, and the latter the case for the E-TMP). This suggests that effects in the TMP were driven by the relational information between sentence subjects and objects, and that these effects were based on the unintentional misattribution of sentenc2e truth values to the Chinese characters. This conclusion suggests that propositions can be both formed or activated under conditions of automaticity [22].

In addition, our findings contribute to the debate relating to the goal (in)dependence of automatic truth evaluation. Specifically, whereas some have argued that automatic truth evaluation is a goal-dependent process [23], others claim that automatic truth evaluation is goal-independent but requires semantic processing in order to occur [32]. Our findings suggest that automatic truth evaluation could occur even in the absence of the goal to process the truth of statements, but that the introduction of goals greatly modulates the probability or extent of automatic truth validation. Specifically, even in the S-TMP (where no truth-evaluation trials were present) we still consistently observed effect sizes in line with our expectations: however, the introduction of the goal of truth evaluation (i.e., a “truth evaluation mindset”) via the inclusion of truth-evaluation trials resulted in substantially-larger effects compared to the those observed in the S-TMP.

One of the most appealing aspects of the TMP is that it carries over the procedural benefits which are typically associated with the AMP and the SMP over other indirect measurement procedures. Specifically, the TMP takes little time to complete (~6 minutes for the S-TMP, ~8 minutes for the E-TMP), is simple in its instructions to participants (though slightly more complex for the E-TMP), does not require relative measurement between items (i.e., the procedure is not arranged in such a way that the endorsement of one belief is examined in the context of its contrast with another belief, as in the case in the IAT, RRT, and IRAP), has extremely high reliability (higher even than the AMP itself), and avoids the high variability which pervades reaction-time-based measures [44]. The TMP, however, also comes with benefits beyond those of the AMP, most notably the ability to capture complex beliefs rather than simple evaluations. In addition, the E-TMP in particular possesses one unique aspect even beyond that of the S-TMP: the ability to capture explicit and implicit beliefs simultaneously in a highly structurally-similar measurement context. That is, the catch trials of the E-TMP require the explicit evaluation of the truth value of the prime sentence, in the same sense that explicit measures of beliefs require. These catch trials are nearly structurally-identical to the implicit trials: the only difference being that a “??-true or false-??” prompt is presented, as opposed to the Chinese character. Such a structural similarity is important because it facilitates the interpretation of divergences between implicit and explicit measures. More specifically, when implicit and explicit measures are structurally similar, it is likely that divergences between the measures reflect actual differences between underlying processes involved in social cognition rather than the mere structural dissimilarity between the measurement procedures being employed [40].

Whereas there are some extant indirect measurement procedures that do bear relatively-close structural similarity to explicit measures (e.g., the qIAT, the aIAT [45, 46]), these measures suffer from one further issue: *quantitative dissimilarity*. That is, measures such as the qIAT (which uses items taken directly from explicit questionnaires) quantify effects in terms of response time differences, whereas explicit measures are typically quantified based on more discrete Likert scale judgements. There is inherent dissimilarity between these quantification methods; for example, response times tend to follow an ex-Gaussian distribution, whereas Likert scales do not [47]. Quantitative dissimilarity may therefore lead to divergences between implicit and explicit measures which are merely the consequence of the method of quantification, rather than based on differences between underlying constructs being assessed.

The E-TMP not only overcomes issues related to structural dissimilarity, but also of quantitative dissimilarity: both implicit and explicit trials are presented within essentially identical procedural contexts and can both be scored based on the proportion of “correct” responses made. Only the operating conditions within each trial type are varied, that is, the goal to evaluate the truth value of either the prime (explicit) or the target (implicit). This bridging of structural and quantitative dissimilarity has formerly been achieved in variations of the AMP [40]. Interestingly, our findings based on the relationships between direct/indirect TMP effects and self-report scores were quite similar to those found by Payne and colleagues. We found that direct and indirect TMP effects correlated at roughly *r* = .51 for “are-vs-should” scores, and *r* = .60 for “men-vs-women” scores, whereas Payne et al. found that direct and indirect AMP effects using Black and White face primes correlated around *r* = .64. Additionally, we found the relationship between indirect TMP effects and self-reports to be *r* = .35 and *r* = .37, whereas Payne et al. found this relationship (indirect AMP effects and self-reports) to be around *r* = .25. The relationship between direct TMP effects and explicit scores, notably, was somewhat larger than the relationship between direct AMP effects and explicit scores (*r* = .43 and *r* = .45, compared to *r* = .25 and *r* = .26). In spite of some variations in specific figures, our pattern of results was consistent with Payne and colleague’s position that direct and indirect AMP effects from within the same procedure correlate highly. Additionally, our results seem to indicate that the correlation between direct TMP effects and self-report scores in higher than the correlation between indirect ratings and self-report scores, which is consistent with the position that direct TMP effects likely reflect explicit evaluations of truth, whereas indirect TMP effects more likely reflect automatic truth evaluations. In this sense, the E-TMP can offer researchers a unique new feature: the ability to explore the implicit-explicit relationship without perturbation from confounding structural/quantitative dissimilarities across measurement procedures.

Whereas the findings across these three experiments are generally supportive of the TMP as a new measure of implicit beliefs, they are not without limitations. One particularly pressing issue relates to the question of intentional use of the primes within the procedure. Although our results provide support for the idea that TMP effects are unintentional, we infer this exclusively through analyses based on self-report measures, which in general are sub-optimal for these purposes [10, 48]. In addition, as one reviewer commented, participants may have difficulty or reticence in reporting that they were intentionally influenced by the prime stimuli for a wide variety of reasons (such as fears about not being paid for failing to follow the perceived demands of the experimenter). For these reasons, the evidence here that TMP effects are driven by unintentional processes is relatively weak and warrants further investigation. Future TMP research should seek to assess this question more thoroughly through the use of non-self-report-oriented analytic strategies, such as observing whether effects persist when participants attend only to task-irrelevant features of the prime stimuli [49] or requiring the evaluation of a response’s (un)intentionality after every trial [10].

One further limitation can found in the fact that, in the E-TMP, the addition of the evaluative catch trials may have resulted in participants pre-emptively preparing evaluative responses to the prime stimuli on some/all trials, including trials where only a response to the target stimulus was required. Given that the response keys for the standard and evaluative trials were identical, the enhanced effect sizes in the E-TMP compared to the S-TMP may be at least in part due to this preparation of responses. Given that participants could only determine the truth value of the sentence upon the presentation of the final word in the prime sentence (and therefore would have little time to prepare any response before knowing which trial type was being presented), we believe this would likely only have minimal (if any) impact in enhancing TMP effects, and that the increased effect size is more likely due to the induction of an evaluative mindset in the participants. Nevertheless, in order to thoroughly rule out this potential confound, future studies using the E-TMP should require participants to use different response keys for the evaluation trials compared to the standard trials.

Beyond the gender stereotypes assessed in the current manuscript, the TMP has prospective use in a variety of other contexts. Implicit measures in general are increasingly often applied in clinical contexts in order to assess cognitions of clinical patients [50, 51, 52, 53]. Whereas associative measures like the IAT have demonstrated utility in some contexts, mainstream cognitive theories of psychopathology frequently emphasise the role of discrepancies between the actual state of an individual, and their idealised/desired state, in the occurrence of psychological disorders and mental distress [54, 55, 56, 57]. In this sense, the use of associative indirect measurement procedures is not sufficient: the measurement of beliefs is required. The TMP therefore provides researchers with the opportunity to assess discrepancies between automatic beliefs, beyond associations, which may provide further insight into this aspect of psychopathology. As mentioned earlier, relational indirect measurement procedures such as the IRAP and the RRT have already demonstrated utility in some such contexts [24, 25, 26, 27]. The TMP may supplement this battery of measures in several ways.

A first advantage is the already-discussed ability of the E-TMP to capture explicit and implicit beliefs simultaneously, in a highly structurally- and quantitatively-similar measurement context. A second advantage is that the TMP operates based on different mechanisms to the RRT and the IRAP, and this confers specific advantages. Given that (i) behaviours may be automatic in some aspects, but not others (e.g., fast, but not unintentional; [58]), (ii) the specific features of automaticity, and their mechanisms, which are present/absent vary across behaviors [59], and (iii) implicit measures should be of greatest predictive utility when the features of automaticity which are captured in the measure overlap with the features of automaticity present in the behavior to be predicted [60, 61], there is a need for a range of well-validated implicit measures that capture different features of automaticity, and operate based on different mechanisms. The TMP as a measure of unintentional misattribution of beliefs, then, can be of greater use than the IRAP or the RRT in specific contexts, more specifically, where to-be-predicted behaviours are related to misattribution (rather than based on response-conflict, as in the IRAP or RRT). On the other hand, the TMP may also supplement research using the IRAP or RRT, by providing researchers with a tool to corroborate findings from these other measures, in order to verify that findings are not merely specific to an individual measurement procedure (in the same way that the AMP is often used to corroborate findings in the IAT; see [8], for an example).

The third advantage is that the TMP is capable of assessing multiple beliefs simultaneously within a single procedure, which the IRAP and RRT currently cannot do. In studies which have formerly used the IRAP or the RRT to assess multiple beliefs, this has been achieved through the completion of multiple versions of the procedure (e.g., first completing an ‘actual’ RRT, and then an ‘ideal’ RRT) [25]. However, this sequential completion of multiple RRTs opens questions relating to effects of practice, as well as being generally more time-consuming for researchers and participants. The ability to capture multiple implicit beliefs simultaneously within the same testing context is therefore an advantage which is, at present, unique to the TMP. As such, the TMP provides unique advantages beyond other implicit measures of beliefs (capturing the automaticity feature of unintentionality; assessing multiple implicit beliefs at once; assessing implicit and explicit beliefs simultaneously). It also has usefulness for verifying that effects in other implicit measure of beliefs are generalizable rather than measure-specific. For these reasons, we believe that the TMP represents a valuable new addition to the implicit measures toolkit.
